# Is Vitamin D Supplementation Useful for Weight Loss Programs? A Systematic Review and Meta-Analysis of Randomized Controlled Trials

**DOI:** 10.3390/medicina55070368

**Published:** 2019-07-12

**Authors:** Simone Perna

**Affiliations:** Department of Biology, College of Science, University of Bahrain, Sakhir Campus P.O. Box 32038, Bahrain; simoneperna@hotmail.it

**Keywords:** obesity, diet, metabolic syndrome, bariatric surgery, diabetes

## Abstract

*Background and Objectives:* The controversy about the impact of vitamin D supplementation on weight loss treatment was observed in several randomized controlled trials (RCTs). This meta-analysis investigates the effects of vitamin D supplementation (cholecalciferol or ergocalciferol) on weight loss through holistic measurements of Body Mass Index (BMI), weight and waist circumference. *Materials and Methods:* Google Scholar, WOS, PubMed and Scopus were explored to collect relevant studies. The selected articles focused on vitamin D supplementation in overweight and obese individuals with different conditions. Eleven RCTs were included into this meta-analysis with a total of 947 subjects, with a mean of the follow-up from 1 to 12 months and different vitamin D interventions (from 25,000 to 600,000 IU/monthly of cholecalciferol). *Results:* The meta-analyzed mean differences for random effects showed that cholecalciferol supplementation deceases the BMI by −0.32 kg/m^2^ (CI95% −0.52, −0.12 kg/m^2^, *p* = 0.002) and the waist circumference by −1.42 cm (CI95% −2.41, −0.42 cm, *p* = 0.005), but does not statistically affect weight loss −0.43 kg (CI95% −1.05, +0.19 kg, *p* = 0.17). *Conclusions:* This meta-analysis lays the foundation for defining the potential clinical efficacy of vitamin D supplementation as a potential therapeutic option for weight loss programs, but further studies are needed to confirm the validity of these findings and delineate potential underlying mechanisms.

## 1. Introduction

Obesity is defined as a complex chronic condition of nutrient accumulation that manifests by abnormal or excessive fat accumulation, and is associated with increased morbidity and mortality [1]. The International Obesity Task Force and the World Health Organization (WHO) report that over 1.1 billion adults worldwide are overweight, 312 million of whom are obese [2].

Obesity is associated with a series of metabolic disorders, such as insulin resistance, hyperinsulinemia, cancer, abnormal fasting glycaemia, symptomatic diabetes mellitus, dyslipidemia and cardiovascular disorders. Being overweight and obesity have been reported to influence respiratory function, skeletal disorders and kidney function and leads to alimentary dysfunction. For example, serum The 25-hydroxy vitamin D 25(OH)VitD levels are negatively correlated with obesity and associated disorders such as non-alcoholic fatty liver disease [3].

In addition, vitamin D is a fat-soluble hormone that plays a crucial role in increasing the intestinal absorption of calcium, magnesium and phosphate. The precursor of vitamin D is cholecalciferol, which is synthetized by 7-dehydrocholesterol in the skin when it is exposed to sunlight, and is hydroxylated first into the liver as 25-hydroxycholecalciferol 25(OH)VitD. Studies have demonstrated that serum 25(OH)VitD concentrations have a negative correlation with body mass index (BMI) and with several anthropometric or biochemical surrogates [4,5].

In addition, a lower 25(OH)VitD status could cause several diseases, including osteoporosis, types of cancer, diabetes, hypertension and cardiovascular disease [6].

Low blood concentration of 25(OH)VitD is very common in subjects characterized by obesity, possibly due to insufficient dietary intake and too small an amount of outdoor physical activity. In addition, the higher the fat mass is, the higher the chance is to have lower blood concentration of 25(OH)VitD, which could result from sequestration of this vitamin into the adipose tissue [7].

A recent review suggested that there are several possible vehicles for the relationship between 25(OH)VitD and weight loss [8]. The lack of 25(OH)VitD status has been thought to be a possible reason behind higher adiposity through the regulation of parathyroid hormone (PTH) and modulation of adipogenesis [8].

As a direct consequence of low 25(OH)VitD levels, there is an increase of PTH that promotes calcium influx into adipocytes, which enhances lipogenesis, causes catecholamine to induce lipolysis and leads to significant fat accumulation and weight gain [9].

Moreover, the 1,25-dihydroxyvitamin D, the active form of vitamin D, can promote and induce apoptosis in adipocytes [10]. Also, the literature suggests that lower PTH levels can cause weight loss via a sympathetic nervous system-mediated thermogenesis and lipolysis [11].

This meta-analysis investigates the effects of vitamin D supplementation (cholecalciferol or ergocalciferol) on weight loss through holistic measurements of BMI, weight and waist circumference.

## 2. Materials and Methods

### 2.1. Search Strategy

English-written articles were identified by searching Google Scholar, WOS, PubMed and Scopus databases. The search strategy was based on the following search terms: vitamin D (MeSH Terms) OR cholecalciferol or ergocalciferol (MeSH Terms) OR vitamin D2 (MeSH Terms) OR calcitriol (MeSH Terms) OR vitamin D3 (MeSH Terms) AND body weight( MeSH Terms) OR body mass index (MeSH Terms)) OR waist circumference (MeSH Terms) OR parathyroid hormone (MeSH Terms) OR 25OHD (MeSH Terms)) OR Anthropometric (MeSH Terms)) OR metabolic syndrome (MeSH Terms) OR fat mass (MeSH Terms).

### 2.2. Study Selection

This meta-analysis was based on the Preferred Reporting Items for Systematic Reviews and meta-analysis guidelines (PRISMA). PRISMA is an evidence-based set of items that can be used as a basis for reporting systematic reviews of research evaluations. A structured approach using five components was adapted in order to construct the research question. These five components are as follows: 1. Participants, 2. Interventions, 3. Comparators, 4. Outcomes, 5. Study design (PICOS).

For each study, the following data were collected: first author, publication year, study setting, study design, eligibility criteria, number of subjects, gender, age, race-country, treatment duration and the main outcomes. A meta-analysis for pooled estimate for aggregated data was performed.

#### 2.3. Participants

We selected overweight and obese adults (BMI ≥25 kg/m^2^, age ≥18 years). No constrains were assigned with regards to gender, diseases, race and geographical distribution of the individuals enrolled in the study.

#### 2.4. Interventions

Randomized clinical trials investigating the effectiveness of vitamin D (cholecalciferol or ergocalciferol) on different anthropometric outcomes were included. The intervention considered the vitamin D oral supplementation as ergocalciferol or cholecalciferol alone or with another intervention such as diet, physical exercise or other supplements, such as calcium.

#### 2.5. Outcomes

Eligible studies were required to report baseline and follow-up values, the mean change (∆-change) and relative standard deviation from baseline and/or the mean difference among intervention groups vs. control group concerning anthropometric outcomes such as body weight, BMI and waist circumference. 

##### 2.5.1. Inclusion and Exclusion Criteria

We included all randomized controlled trials on humans and conducted within the last 10 years. We excluded from the selection non–English-language animal studies, in vitro studies, non-RCTs in overweight and obese patients and RCTs in adults with BMI <25kg/m^2^.

Studies in children or studies that were not randomized or had no control group were excluded a priori.

##### 2.5.2. Risk of Bias in Individual Studies

The risk of bias of each study was assessed using the Cochrane Collaboration using the Risk of Bias tool [12] and considering factors contributing to the study quality, the generation of the allocation sequence, the allocation concealment, the blinding of outcome data, the presence of incomplete data and the selective reporting.

These factors were classified as low risk of bias, high risk of bias or unclear risk of bias. Studies with a low risk of bias for at least three items were held as good, studies with a low risk of bias for at least two items were considered as fair and studies with a low risk for no item or only for one item were regarded as poor.

## 3. Results

**Figure 1:**
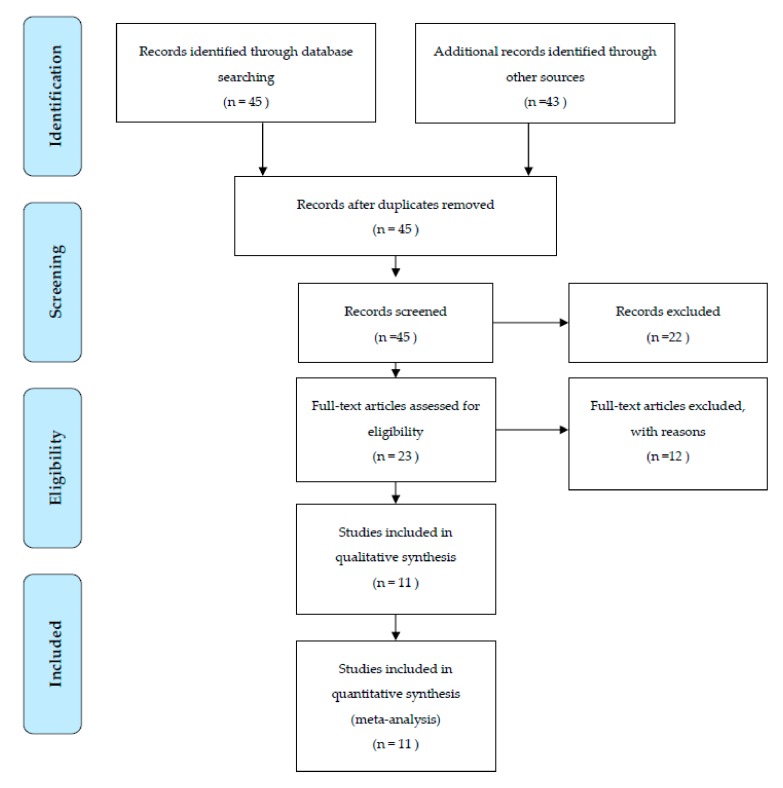
Flow chart.

The literature search, after the initial screening, retrieved 23 eligible articles. After an accurate screening, 19 papers were selected for full-text revision. Of the remaining 8 articles, 10 studies were excluded for methodological reasons and 11 RCT studies were selected for the present systematic review and meta-analysis. Figure 1 shows the study selection procedure.

Table 1 summarizes the studies selected for this meta-analysis. It worth nothing that the geographical location for each study was different. In addition, cholecalciferol was the only form of vitamin D supplementation used among the selected studies. Concerning the study design, 11 articles were double-blind RCTs, the intervention lasted from 1 to 12 months and the range of age of the population was from 18 to 70 years. Eleven studies were included with a total of 947 subjects, both women and men (Table 1).

The control groups were set to consume a hypocaloric diet alone or with physical activity or calcium, without supplements of cholecalciferol and with or without calcium supplement only. The intervention groups were supplemented with cholecalciferol alone (at dose from 25,000 IU to 600,000 IU/monthly) or in combination with calcium (dose of 150, 170, 250, 500 or 800 mg).

The primary outcome was the assessment of the weight loss. Secondary outcomes included BMI and waist circumference. As showed in Table 1, among the 11 RCT articles that examined the relationship between cholecalciferol intake and BMI, six articles reported a significant positive association, while only two reported no statistically significant effect. Two studies combined the intervention of cholecalciferol supplementation with an exercises program and two other studies assessed the calcium intervention in addition to cholecalciferol supplementation. One article investigated the effectiveness of cholecalciferol supplementation with another combined intervention using a hypo-caloric diet, while the remaining six articles used only cholecalciferol supplement compared to a placebo.

### 3.1. Meta-Analyzed Data

As reported in Table 2, the meta-analyzed mean differences for random effects (MD) showed a statistically significant decrease in BMI −0.32 kg/m^2^ (CI95% −0.52, −0.12 kg/m^2^) The test for overall effect was Z: 3.09 (*p* = 0.002). The heterogeneity was I^2^ = 66%.

As showed in Table 3, cholecalciferol supplementation decreased body weight in four studies, whereas two reported no association. Meta-analysis has shown that cholecalciferol supplementation does not affect weight loss. The mean difference in body weight across all the studies was −0.43 kg (CI95% −1.05, 0.19 kg) (*p* = 0.17) The test for overall effect was Z: 1.36 (*p* = 0.0005). The heterogeneity was I^2^ = 77%.

In Table 4, three studies with cholecalciferol supplementation showed a decrease in waist circumference over the course of the experiment, while only one study demonstrated an increase in waist circumference in the cholecalciferol group. The meta-analyzed random effects mean differences (MD) showed a statistically significant decrease in waist circumference of 1.42 cm (CI95% –2.41, –0.42 cm) and the average effect size was 2.79 (*p* = 0.005). The heterogeneity was I^2^ = 80%.

### 3.2. Risk of Bias

No publication bias was indicated for any outcomes, as determined via a funnel plot inspection and Begg’s and Egger’s test *p*-values >0.05 (Table 5).

## 4. Discussion

The systematic review and meta-analysis of 11 RCTs with more than 900 subjects lasting between 1 to 12 months after cholecalciferol supplementation (from 25,000 to 600,000 IU monthly), revealed that cholecalciferol has a desirable effect on weight loss by reducing BMI and waist circumference in overweight and obese individuals, while no data was found on waist hip ratio and the other body compositions, such as fat mass and fat percentage.

From a theoretical point of view, there are many mechanisms reported in the literature that claim to explain the process through which cholecalciferol supplementation supports the weight loss process [17].

A recent study has reported that cholecalciferol has physiological and biochemical effect in a way that reduces metabolic abnormalities and tissue damage that can result from adiposity [7].

Cholecalciferol has a direct role in suppressing the PTH hormone, which promotes and triggers fat accumulation in the adipose tissue via increasing intracellular calcium [5].

A recent study reported that cholecalciferol supports intestinal calcium absorption which assists in weight loss [6]. Another suggested mechanism states that cholecalciferol stimulates insulin receptors and is responsible for maintaining the calcium homeostasis, which is an important factor for intracellular mediated processes. Increasing body size is the accumulative consequence of the claimed association between cholecalciferol and insulin resistance [10].

Some studies showed that taking Ca supplements in addition to cholecalciferol increased the inverse relationship and decreased the fat mass that was attributed to calcium metabolism. It is believed that a calcium-rich diet increases fat oxidation, promotes fat cell apoptosis and decreases lipid absorption through the process of insoluble calcium-fatty acid soap formation in the intestine. In addition, the presences of calcium in a diet leads to suppressing 25(OH)VitD levels, which in turn decreases the calcium influx into the cell and eventually triggers the lipolysis process and suppresses lipogenesis in the adipocyte [18,19].

It is noteworthy to highlight and emphasize the results of this meta-analysis and apply them in clinical practice. 25(OH)VitD levels must be taken into consideration as a strong factor that contributes to weight gain among overweight and obese people. Prescribing cholecalciferol and following an effective strategy for cholecalciferol supplementation should be an imperative practice, especially for overweight and obese individuals

One of the main strengths in this meta-analysis is the design of the studies that were analyzed, as all used randomized, placebo-controlled designs that led to causative conclusions.

In addition, this meta-analysis investigated the effect of vitamin D on weight loss using a comprehensive approach with holistic anthropometric measurements of percentage and fat mass to measure the differences before and after intervention. This approach did not only depend on just a single variable, which enhances and promotes the study characteristics. Moreover, one article discussed the effect of cholecalciferol combined with another intervention, such as dietary control, exercise, or calcium intake, which makes it difficult to make conclusive remarks to distinguish an independent vitamin D supplementation clinical effect for weight loss purposes [19].

Moreover, few clinical trials of cholecalciferol supplementation on body composition were done using a combination of calcium and cholecalciferol supplementation. Previous studies have demonstrated that cholecalciferol supplementation positively influences BMI, weight and waist circumference [15,16,20].

However, the adequate dosages and duration of cholecalciferol supplementation are still unclear. Therefore, further extensive clinical trials involving larger sample sizes are required to evaluate whether cholecalciferol supplementation has an effect on another anthropometric outcomes, such as waist to hip ratio and changes in body composition.

Several limitations of the present study should be noted. First, the number of eligible studies was small. We evaluated the efficacy of cholecalciferol supplementation on weight loss and anthropometric outcomes independent of researcher-imposed energy restriction or weight-loss counseling. Finally, other potential additional factors such as diet, follow up, cholecalciferol dosage and physical activity were different among the RCTs.

## 5. Conclusions

This meta-analysis lays the foundation for defining the potential clinical efficacy of cholecalciferol D supplementation as a potential therapeutic option for weight loss programs, but further studies are needed to confirm the validity of these findings and delineate the potential underlying mechanisms, the correct dosage responsiveness and the timing of supplementation.

## Figures and Tables

**Table 1:** Characteristics of the studies.

First Author/Year of Publication	Subjects, Gender (Cholecalciferol vs. Placebo)	Race (Country)	Population (Age, BMI)	Treatment Duration	Vitamin D Dosage	Outcomes
Salehpour et al., 2012	Total: 85 (85 F) (42 vs. 43)	Arabic (Iran)	18–50 years old, a BMI ≥25 kg/m^2^	12 weeks	1000 IU/ daily as cholecalciferol	Weight, BMI and Waist circumference
Khosravi et al., 2018	Total: 55 (55 F) (26 vs. 27)	Arabic (Iran)	20–40 years females, BMI higher than 25 (obese and overweight)	6 weeks	50,000 IU/weekly cholecalciferol	Weight, BMI, Waist circumference
Mason et al., 2014	Total: 218 (218 F) (109 vs. 109)	Non-Hispanic white n = 188; Non-Hispanic black n = 13; Hispanic n = 5; Other n = 12 (USA)	Healthy women aged 50–75 years who were overweight or obese BMI (in kg/m^2^)	12 months	2000 IU/daily oral cholecalciferol + weight-loss program	Weight, Waist circumference
Cefalo et al., 2018	Total: 18(14 F, 4 M) (9 vs. 9)	White European (Italy)	Male and female subjects aged 18 to 70 years with BMI 30 kg/m^2^	3 months	25,000 IU/ weekly cholecalciferol + hypocaloric diet	Weight, BMI
Zittermann et al., 2009	Total: 165 (135 M, 30 F) (82 vs. 83)	White European (Germany)	Healthy overweight age of 18–70 years and a BMI >27 kg/m^2^	12 months	800 IU/daily oral cholecalciferol	Weight, BMI, Waist circumference
Duggan et al., 2015	Total: 218 (218 F) (93 vs. 94)	Non-Hispanic white n = 188 Non-Hispanic Black n = 13 Hispanic n = 5; Other n = 12 (USA)	Postmenopausal, overweight/obese (BMI ≥25 kg/m^2^), ages 50–75 years)	12 months	2000 IU/ daily cholecalciferol + weight-loss intervention	BMI
Esmaeil et al., 2018	Total: 58 (58 F) (30 vs. 28)	Arabic (Iran)	Type-2 diabetic patients aged 30 to 60 years, BMI over 25 kg/m^2^	2 months	4000 IU/ daily cholecalciferol	Weight, BMI, Waist circumference
Shapses et al., 2013	Total: 41 (41F) (19 vs. 22)	Hispanic n = 41 (USA)	aged between 50 and 70 years (BMI ≥25 kg/m^2^)	6 weeks	2500 IU/ daily oral cholecalciferol	Weight
Mai et al., 2018	Total: 24 (13 M, 11 F) (12 vs. 12)	White European (Italy)	Female and male age 38 ± 2.4 years, BMI 42.7 ± 1.3 kg/m^2^	4-weeks	600,000 IU/monthly cholecalciferol	Weight and BMI
Majoret et al., 2018	Total: 13 (13F) (7 vs. 6)	White European (UK)	obese women BMI ≥30 (mean age 43 years, mean BMI 32 kg/m^2^)	15-weeks	600 mg elemental calcium + 400 IU/daily cholecalciferol	Weight, waist circumference
Zhu et al., 2013	Total: 52 (13M, 39F) (26 vs. 26)	Asian (China)	Healthy overweight or obese (BMI) of BMI ≥25 kg/m^2^, aged 18 to 25 years	12 weeks	600 mg of calcium carbonate plus 125 IU cholecalciferol/ daily	Weight

* F: females; M: males; IU: International Unit.

**Table 2:** Effect of cholecalciferol supplementation on body mass index (BMI).

Study	Cholecalciferol			Placebo				Total
	Mean	SD	Total	Mean	SD	Total	Weight	Mean Difference iv Random, CI95%
Esmaeil et al., 2018	−0.4	0.11	30	−0.06	0.1	28	34.9%	**−0.34 (−0.39, −0.29)**
Khosravi et al., 2018	−0.61	0.5	26	0.02	0.5	27	21.9%	**−0.63 (−0.9, −0.36)**
Salehpour et al., 2012	−0.13	0.6	42	−0.04	0.6	43	22.8%	**−0.09 (−0.35, 0.17)**
Mai et al., 2018	−6	0.7	12	−5.5	0.5	12	11.6%	**−0.5 (−0.99, −0.01)**
Zitterman et al., 2009	−2	2	82	−2.2	1.9	83	8.7%	**0.2 (−0.4, 0.8)**
Total (CI95%)			320			322	100%	**−0.32 (−0.52, −0.12)**

* Bold represents Mean Differences (MD) and CI95%.

**Table 3:** Effect of cholecalciferol supplementation on body weight.

Study	Cholecalciferol			Placebo				Total
	Mean	SD	Total	Mean	SD	Total	Weight	Mean Difference iv Random, 95%CI
Zitterman et al., 2009	−5.7	5.8	82	−6.5	5.6	83	8.3%	**0.8 (−0.94, 2.54)**
Shapses et al., 2013	−3	1.2	19	−3.3	1.2	22	17.9%	**0.3 (−0.44, 1.04)**
Salehpour et al., 2012	−0.3	1.5	42	−0.1	1.7	43	18.6%	**−0.2 (−0.88, 0.48)**
Mai et al., 2018	−5.8	0.4	12	−5.5	0.5	12	22.1%	**−0.3 (−0.66, 0.06)**
Esmaeil et al., 2018	−1.1	0.31	30	−0.15	2.9	28	13.9%	**−0.95 (−2.03, 0.13)**
Khosravi et al., 2018	−1.6	1.3	26	0.05	1	27	19.2%	**−1.65** **(−2.28,** **−1.02)**
Total (CI95%)			362			365	100%	**−0.43 (−1.05, 0.19)**

* Bold represents Mean Differences (MD) and CI95%.

**Table 4:** Effect of cholecalciferol supplementation on waist circumference.

Study	Cholecalciferol			Placebo				Total
	Mean	SD	Total	Mean	SD	Total	Weight	Mean Difference iv Random, 95%CI
Esmaeil et al., 2018	−1.51	0.48	30	−0.05	0.5	28	38.1%	**−1.46 (−1.71, −1.21)**
Khosravi et al., 2018	−2.3	1	26	0.3	1.5	27	32.9%	**−2.6 (−3.28, −1.92)**
Salehpour et al., 2012	−0.3	4.3	42	0.4	4.1	43	17.3%	**−0.7 (−2.49, 1.09)**
Zitterman et al., 2009	−6.5	9.6	82	−7.5	5.8	83	11.7%	**1 (−1.42, 3.42)**
Total (95%CI)			296			296	100%	**−1.42 (−2.41, −0.42)**

* Bold represents Mean Differences (MD) and CI95%.

**Table 5:** Bias for studies included in the meta-analysis according to the Cochrane Risk of Bias Tool ^a^.

Study, Year (Ref No.)	Random-Sequence Generation	Allocation Concealment	Participant-Personnel Blinding	Outcome-Assessment Blinding	Incomplete Outcome Data	Selective Reporting	Other Bias
Cefalo et al., 2018 [1]	Low	High	Low	Unclear	Low	Low	Low
Duggan et al., 2015 [3]	Low	Unclear	Low	Unclear	Low	Low	Low
Esmaeli et al., 2018 [4]	Low	Unclear	Low	Unclear	Low	Low	Low
Shapses et al., 2013 [5]	Unclear	Unclear	Low	Unclear	Low	Low	Low
Salehpour et al., 2012 [7]	Low	High	Low	Unclear	Low	Low	Low
Khosravi et al., 2018 [10]	Low	Low	Low	Unclear	Low	Low	Low
Zhu et al., 2013 [11]	Low	Low	Low	Unclear	Low	Low	Low
Mason et al., 2014 [13]	Low	Unclear	Low	Unclear	Low	Low	Low
Mai et al., 2018 [14]	Low	Unclear	Low	Unclear	Low	Low	Low
Zittermann et al., 2009 [15]	Unclear	Unclear	Low	Unclear	Low	Low	Low
Major et al., 2008 [16]	Low	Low	Low	Unclear	Low	Low	Low

^a^ Bias designations by study criteria are indicated by seven domains with categories including low risk if negative aspects of the study design were not likely to influence the study findings, high risk if the study design was likely to influence the study findings or unclear risk if high or low risk could not be assigned because of a lack of evidence.
